# Convergent evolution of pathogenicity islands in helper *cos* phage interference

**DOI:** 10.1098/rstb.2015.0505

**Published:** 2016-11-05

**Authors:** Nuria Carpena, Keith A. Manning, Terje Dokland, Alberto Marina, José R. Penadés

**Affiliations:** 1 Institute of Infection, Immunity and Inflammation, College of Medical, Veterinary and Life Sciences, University of Glasgow, Glasgow G12 8TA, UK; 2 Departamento de Ciencias Biomédicas, Facultad de Ciencias de la Salud, Universidad CEU Cardenal Herrera, 46113 Moncada, Valencia, Spain; 3 Department of Microbiology, University of Alabama at Birmingham, Birmingham, AL 35294, USA; 4 Instituto de Biomedicina de Valencia (IBV-CSIC) and CIBER de Enfermedades Raras (CIBERER), 46010 Valencia, Spain

**Keywords:** capsid morphogenesis, SaPIs, PICIs, small capsids, bacteriophage resistance, bacteriophage packaging

## Abstract

*Staphylococcus aureus* pathogenicity islands (SaPIs) are phage satellites that exploit the life cycle of their helper phages for their own benefit. Most SaPIs are packaged by their helper phages using a headful (*pac*) packaging mechanism. These SaPIs interfere with *pac* phage reproduction through a variety of strategies, including the redirection of phage capsid assembly to form small capsids, a process that depends on the expression of the SaPI-encoded *cpm*A and *cpm*B genes. Another SaPI subfamily is induced and packaged by *cos*-type phages, and although these *cos* SaPIs also block the life cycle of their inducing phages, the basis for this mechanism of interference remains to be deciphered. Here we have identified and characterized one mechanism by which the SaPIs interfere with *cos* phage reproduction. This mechanism depends on a SaPI-encoded gene, *ccm*, which encodes a protein involved in the production of small isometric capsids, compared with the prolate helper phage capsids. As the Ccm and CpmAB proteins are completely unrelated in sequence, this strategy represents a fascinating example of convergent evolution. Moreover, this result also indicates that the production of SaPI-sized particles is a widespread strategy of phage interference conserved during SaPI evolution.

This article is part of the themed issue ‘The new bacteriology’.

## 1. Introduction

The *Staphylococcus aureus* pathogenicity islands (SaPIs) are the prototypical members of a novel family of mobile genetic elements, the phage-inducible chromosomal islands (PICIs). These elements are intimately related to certain helper phages, whose life cycles they parasitize [1], driving helper phage evolution [2]. Following infection by a helper phage or SOS induction of a helper prophage, the PICI genome excises, using the PICI-encoded integrases (*int*) and excision functions (*xis*) [3,4]. The PICI genome replicates extensively using its replicon [5,6] and is efficiently packaged into infectious particles composed of phage-encoded structural proteins [7,8]. These events, which constitute the excision–replication–packaging (ERP) cycle of the PICIs, allow both the intra- and intergeneric transfer of these elements at extremely high frequencies [9,10]. The hallmark of this parasitism is a key PICI gene that encodes a master repressor (Stl), which controls expression of most of the PICI genome. Contrary to the classical phage repressors, the Stl repressors are not cleaved following activation of the SOS response; rather the repression is lifted by the formation of a complex between the repressor and a specific helper phage protein [11,12], thereby linking PICI replication to the helper phage lytic cycle.

**Figure 1.**
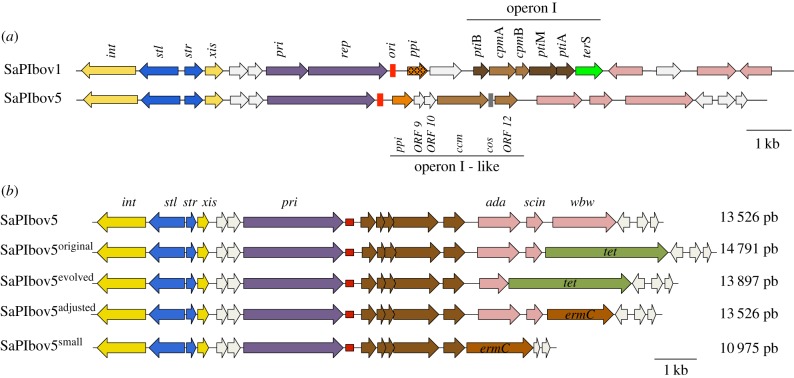
Genomic structure of the *cos* SaPIs. (*a*) Comparison of the *pac* (SaPIbov1) and *cos* (SaPIbov5) SaPIs. (*b*) Alignment of selected SaPIbov5 size adjustment. Genomes are aligned according to the prophage convention with the integrase gene at the left end. Gene colour code: *int* and *xis*, yellow; transcription regulators, blue; replication genes, purple; replication origin, red; genes affecting expression (*pti*) or assembly (*cpm*) of helper phage virion components are dark brown and medium brown, respectively; the terminase small subunit gene (*ter*S) is green; *pip* (phage interference) orange, the two variant subsets are distinguished by dark versus light fill; superantigen and other accessory genes, pink. Genes encoding hypothetical proteins, white. In (*a*), the *cos* site is shown in grey. In (*b*), the tetracycline resistance gene is light green, and the erythromycin resistance gene is dark red.

Another key feature of all the analysed PICIs is their capacity to severely interfere with phage reproduction. To date, all described mechanisms of phage interference target key proteins of the phage DNA packaging machinery. Like their helper phages, PICIs can be packaged using two different strategies: a headful (also called *pac*) mechanism, in which DNA packaging continues until the capsid is full; or *cos* site packaging, in which units of DNA delimited by *cos* sites are packaged [13]. Most of the characterized SaPIs (and their helper phages) use the headful packaging mechanism for packaging. The *pac* SaPIs encode a small terminase subunit (TerS_SP_) which interacts with the phage-coded large terminase subunit (TerL), promoting SaPI-specific DNA packaging [14,15]. Additionally, many *pac* SaPIs redirect the helper phage assembly pathway to generate SaPI capsids that are one-third of the size of the helper phage capsids [16,17], commensurate with the smaller size of the SaPI genome. The small SaPI capsids are incapable of accommodating complete helper phage genomes [17–19]. This size redirection depends on the SaPI-encoded *cpm*A and *cpm*B genes [5,20–22]. Like *ter*S_SP_, the *cpm*AB genes are located in the SaPI packaging module, also termed operon I (figure 1), whose expression is controlled by the SOS-specific repressor LexA [14]. Apparently, the *raison d’être* of this operon is to interfere with phage reproduction. Operon I also contains the *pti*A, *pti*B and *pti*M genes (figure 1) [23]. PtiA and PtiM modulate the function of the late phage gene transcriptional regulator LtrC [23–25], while the mechanism of phage interference depending on PtiB remains unresolved [23]. The remaining known mechanism of interference depends on the *ppi* gene, located between the SaPI *ori* site and the SaPI packaging module (figure 1). The SaPI-coded Ppi protein interacts with the phage TerS, preventing phage DNA packaging [26].

We recently identified a subfamily of SaPIs in which the complete operon I, except the 3′ region of the SaPI *ter*S_SP_ gene, had been replaced by a DNA region, that we have termed ‘operon I-like’, containing a highly conserved phage *cos* site (electronic supplementary material, figure S1) and a set of conserved genes whose functions remain obscure (figure 1). These variants, represented by SaPIbov4 and SaPIbov5 [27], are induced by certain *cos* phages, such as ϕ12 or ϕSLT, which all share basically the same *cos* site (electronic supplementary material, figure S1), and are efficiently packaged in infectious phage-like particles, leading to high-frequency intra- and intergeneric transfer [9,28]. While these variant islands lack the classical operon I, they also severely interfere with phage reproduction [28], suggesting they encode alternative strategies of phage interference. In this report we characterize the first interference mechanism involving *cos* SaPIs and show that these SaPIs also redirect the capsid assembly of their helpers using a novel mechanism.

## 2. Material and methods

### (a) Bacterial strains and growth conditions

The bacterial strains used in this study are listed in the electronic supplementary material, table S1. The procedures for preparation and analysis of phage lysates, in addition to transduction and transformation of *S. aureus*, were performed essentially as previously described [11,12,18].

#### (i) DNA methods

General DNA manipulations were performed using standard procedures. DNA samples were heated at 75°C for 10 min prior to the electrophoresis to ensure *cos* site melting. The plasmids and oligonucleotides used in this study are listed in the electronic supplementary material, tables S2 and S3, respectively. The labelling of the probes and DNA hybridization were performed according to the protocol supplied with the PCR-DIG DNA-labelling and Chemiluminescent Detection Kit (Roche). To produce the phage and SaPI mutations, we used plasmid pBT2-βgal, as previously described [11].

#### (ii) Complementation of the mutants

The different phage genes under study were PCR amplified using oligonucleotides listed in the electronic supplementary material, table S3. PCR products were cloned into pCN51 [29] and the resulting plasmids (electronic supplementary material, table S2) were introduced into the appropriate recipient strains (electronic supplementary material, table S1).

### (b) Experimental evolution

A ϕSLT lysogen carrying the SaPIbov5 *tet*M island was SOS (mitomycin C) induced and the island transferred to a ϕSLT lysogen. After the transfer, the SaPIbov5-positive strains were recollected and the procedure repeated four more times. After the fifth passage, three individual colonies were isolated, SOS induced and the SaPI titre obtained compared with that obtained with the original SaPIbov5 *tet*M.

### (c) Electron microscopy

To produce ϕ12 phage and SaPIbov5 transducing particles, strains JP10435 and JP12419, respectively, were induced with 1 mg l^−1^ mitomycin C at OD_600_ = 0.5, and grown for an additional 3 h. As lysis was incomplete, the cell pellets were treated with lysostaphin before collecting lysate supernatants, which were further purified by PEG precipitation and CsCl centrifugation, as previously described [30]. The purified phage and transducing particles were negatively stained with 1% uranyl acetate and observed in an FEI Tecnai F20 electron microscope operated at 200 kV with magnifications of 65 500× or 81 200×. Images were captured on a Gatan Ultrascan 4000 CCD camera.

#### (i) *In silico* protein modelling and structure comparison

The three-dimensional homology models of ϕ12 gp33 and SaPIbov5 Ccm were constructed using the RaptorX (default mode) [31] and Phyre2 (intensive mode) [32] servers. Both servers generated models with low confidence for the N-terminal portions and high confidence for the C-terminal portions of ϕ12 gp33 and SaPIbov5 Ccm (electronic supplementary material, tables S4 and S5). The models of the C-terminal portions of gp33 and Ccm were structurally aligned with Mustang [33] and this alignment was rendered with ESPript v. 3.0 [34].

## 3. Results

### (a) SaPIbov5 is packaged in small capsids

In previous work, we noted that both *cos* phages ϕSLT and ϕ12 induce SaPIbov5 replication to a similar extent, although SaPIbov5 transfer by ϕ12 was approximately 10^2^ times higher than that observed for phage ϕSLT [28]. As the SaPIbov5 *cos* site is more similar to that present in ϕ12 (electronic supplementary material, figure S1), we speculated that this would be the reason underlying the different SaPIbov5 packaging efficiency observed with these two phages. Indeed, when SaPIbov5 was evolved through five cycles of induction in the presence of ϕSLT, the transducing titre increased by up to 10^3^-fold (electronic supplementary material, table S6), indicating that the evolved SaPIs could be efficiently packaged by phage ϕSLT. However, the SaPI *cos* site sequence remained invariable. Instead, the evolved SaPIs had reduced their size by losing some of the virulence genes contained in the island (figure 1). When we originally introduced *tet*M into SaPIbov5, we had artificially increased the size of the element. The evolved SaPI had been restored to its original size. The increased size caused the reduced transfer observed for SaPIbov5.

This restriction on genome size suggested that the *cos* SaPIs, similar to the previously described *pac* SaPIs [16,17], were packaged into capsids smaller than those normally made by the phage, as the helper phage genomes are about 3× larger (42–45 kb) than the SaPI genomes (≈14 kb, figure 1). This is consistent with the *cos* site packaging mechanism, which packages DNA units delimited by *cos* sites at either end [13].

**Figure 2.**
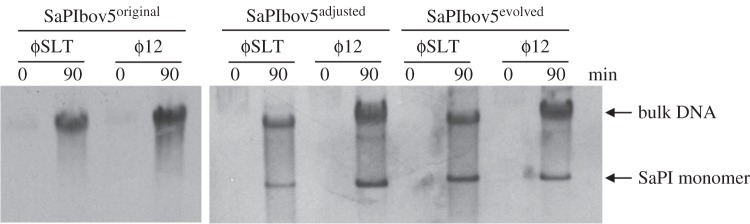
Replication analysis of the different SaPIbov5 derivative islands. Southern blot of ϕ12 and ϕSLT lysates, from strains carrying SaPIbov5^original^, SaPIbov5^adjusted^ and SaPIbov5^evolved^ as indicated (see text for details). Samples were isolated 0 or 90 min after induction with mitomycin C, separated on agarose gels and blotted with a SaPIbov5-specific probe. Upper band is ‘bulk’ DNA, and represents replicating SaPIbov5. SaPI monomer represents SaPI DNA packaged in small capsids.

To test this possibility, we used both the original SaPIbov5 island (SaPIbov5^original^) and the evolved one (SaPIbov5^evolved^), each carrying the *tet*M marker. We also generated a third SaPIbov5 that maintained its correct size but in which part of the *vwb* gene was replaced by an *erm*C marker (SaPIbov5^adjusted^, figure 1). The *vwb* gene encodes the von Willebrand binding protein, a virulence factor with no role in the ERP cycle of the SaPIs [27]. All these islands were introduced into strains LUG1170 and JP10435, lysogenic for the *cos* phages ϕSLT and ϕ12, respectively, and the SaPIbov5 cycle was induced. Remarkably, the evolved and size-adjusted SaPIbov5 islands, but not SaPIbov5^original^, generated the characteristic SaPI-specific band after induction of these islands by phages ϕSLT and ϕ12 (figure 2). All SaPIs, except for the original SaPIbov5^original^, were also highly transferred by these phages (electronic supplementary material, table S6), confirming that the limitation of the SaPI genome size to less than around 14 kb was a prerequisite for high-frequency SaPI transfer.

**Figure 3.**
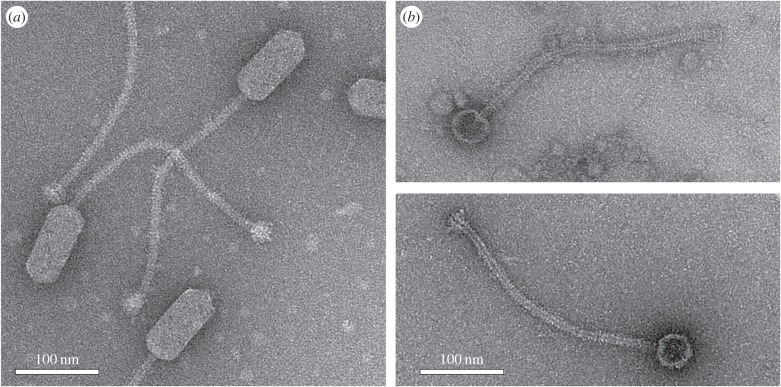
Electron microscopy of ϕ12 and SaPIbov5 particles. Electron micrographs of negatively stained wt ϕ12 virions (*a*), and particles produced by induction of a ϕ12 lysogen containing SaPIbov5^adjusted^ (*b*). Scale bars are 100 nm.

The previous results showed that the length of DNA isolated from capsids produced in the presence of SaPIbov5 was consistent with a single unit of SaPIbov5 DNA, suggestive of formation of small capsids. To confirm that this was the case, we subjected the particles produced by ϕ12 in the absence and presence of SaPIbov5 to electron microscopy (EM). ϕ12 phage particles had the characteristic size and shape of this class of bacteriophages [28]: a prolate head, 45 nm wide and 100 nm long, and a 325 nm long, flexuous tail (figure 3). By contrast, virions produced in the presence of SaPIbov5 had small, isometric heads, about 42–45 nm in diameter, attached to a 325 nm tail (figure 3). This result showed that SaPIbov5 caused the formation of small capsids, consistent with its smaller genome size.

### (b) Identification of the SaPIbov5-encoded capsid size redirection protein

**Figure 4.**
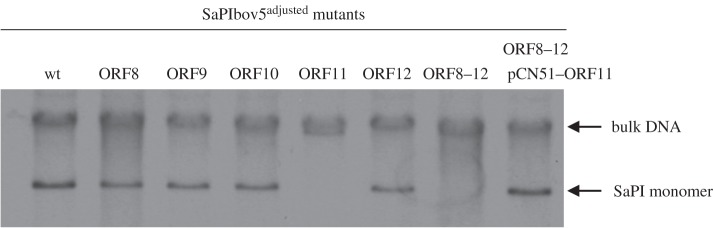
Replication analysis of SaPIbov5 mutants. Southern blot of ϕ12 lysates, from strains carrying the wt or the different SaPIbov5 mutants (carrying ochre mutations in the SaPIbov5 genes 8–12). Samples were isolated 90 min after induction with mitomycin C, separated on agarose and blotted with a SaPIbov5-specific probe. Upper band is ‘bulk’ DNA, and represents replicating SaPIbov5. SaPI monomer represents SaPI DNA packaged in small capsids. SaPIbov5 ORF11 corresponds to *ccm*.

As mobile genetics elements show synteny, and as in *pac* SaPIs the genes involved in phage interference are located between the SaPI *ori* site and the virulence genes, we speculated that the *cpm*-like gene(s) would be located in a similar position in the SaPIbov5 genome. This putative region comprises five genes (operon I-like genes: open reading frames (ORFs) 8–12; figure 1), including *ppi* (SaPIbov5 ORF8) and SaPIbov5 ORF12, which encodes a highly homologous protein (35% identity) to the SaPIbov1 coded PtiM. Both the Ppi and the PtiM have been previously involved in phage interference [23–26]. To identify the gene(s) involved in the formation of the SaPIbov5 small capsids, we generated individual mutants in all the aforementioned five genes by introducing a stop codon (ochre mutation) in the middle of their coding sequences. This strategy does not change the SaPIbov5 size. The different SaPIbov5 mutant islands were then introduced into the ϕ12 lysogen and the SaPIbov5 ERP cycle analysed after SOS induction of the different strains. As shown in figure 4, all the SaPIbov5 mutants except that for SaPIbov5 ORF11 generated the characteristic SaPI-size DNA band on an agarose gel.

We also generated a SaPIbov5 mutant carrying stop codons in all the genes from ORFs 8–12. As expected, this mutant did not generate the characteristic SaPI band when induced by phage ϕ12 (figure 4). However, complementation of this strain with a plasmid expressing ORF11 restored the production of the SaPI characteristic band, confirming the role of ORF11 in capsid size redirection. As the protein encoded by ORF11 seemed to remodel the capsid size of the helper phage, it was renamed Ccm for *cos* capsid morphogenesis.

### (c) Ccm blocks ϕ12 reproduction

**Figure 5.**
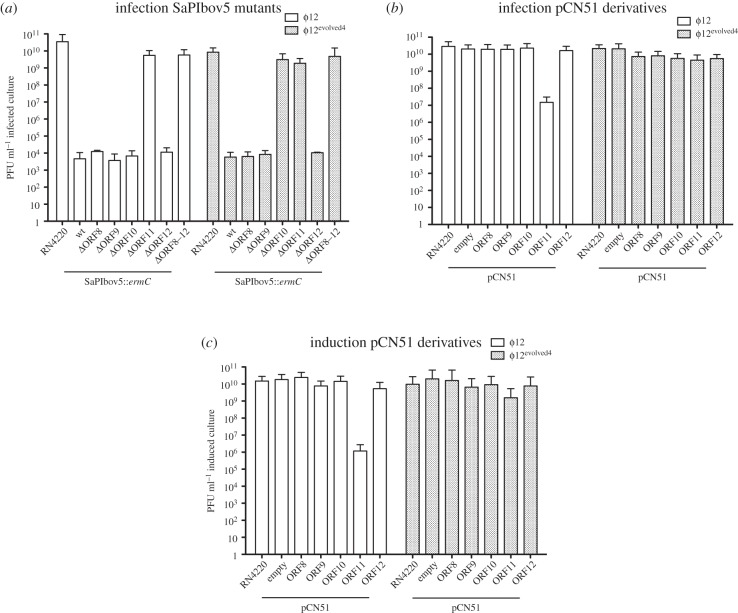
SaPIbov5 Ccm-mediated interference. (*a*) Strain RN4220 containing wt or the different SaPIbov5 mutants were infected with ϕ12 or ϕ12^evolved4^, plated on phage bottom agar, and incubated for 48 h at 32°C. (*b*) Phage interference mediated by cloned SaPIbov5 genes. The indicated genes were cloned into plasmid pCN51. Strain RN4220 containing the indicated plasmids was infected with phages 12 or ϕ12^evolved4^, plated on phage bottom agar containing 5 µM CdCl2 (induces the expression of the cloned genes) and incubated for 48 h at 32°C. (*c*) Effect of the different pCN51 cloned genes in phage reproduction. The lysogenic strains for ϕ12 or ϕ12^evolved4^, containing the different pCN51 derivative plasmids, were SOS induced and the lysates plated on phage bottom agar for 48 h at 32°C.

In previous work, we had demonstrated that SaPIbov5 interferes with ϕ12 reproduction [28]. To test whether this interference was mediated by Ccm, we used two complementary strategies: first, we introduced into the non-lysogenic RN4220 strain the SaPIbov5 mutants described above, including mutants in ORFs 8–11 (*ccm*) and 12 individually and all (ORFs 8–12) together. Then, the capacity of these strains to block plaque formation by phage ϕ12 infection was tested. As shown in figure 5*a*, all mutants except those in the *ccm* gene led to a 10^6^- to 10^7^-fold reduction in ϕ12 titre, showing that Ccm was primarily responsible for the SaPIbov5-mediated interference. Although the number of plaques obtained in the *ccm* mutant was basically the same as in the SaPIbov5-negative strain, the size of the plaques was reduced. This result suggested that some of the other genes may also be involved in phage interference, although this residual effect was not observed when the different genes were analysed individually (figure 5*a*).

Second, SaPIbov5 genes ORFs 8–12 were expressed from the vector pCN51 [29] under control of the exogenous cadmium-inducible promoter P*cad* in the non-lysogenic strain RN4420, followed by infection with ϕ12, or in the ϕ12 lysogen JP10435, followed by SOS induction. In either case, the resulting titres were reduced 10^3^- to 10^4^-fold only upon expression of *ccm* (figure 5*b,c*).

### (d) Target for Ccm-mediated interference

**Figure 6.**
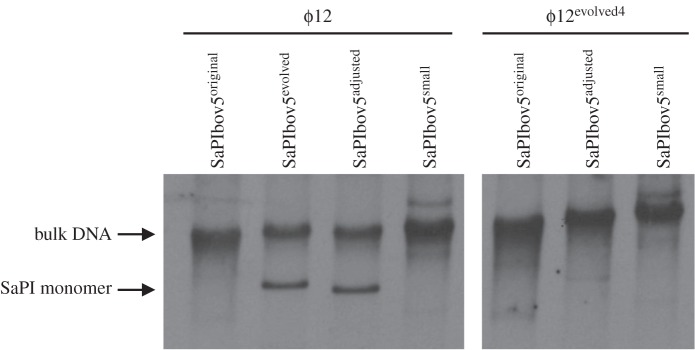
Replication analysis of the different sized SaPIbov5 islands induced by phages ϕ12 or ϕ12^evolved4^. Southern blot of ϕ12 and ϕ12^evolved4^ lysates, from strains carrying SaPIbov5^original^, SaPIbov5^evolved^, SaPIbov5^adjusted^ or SaPIbov5^small^, as indicated. Samples were taken 90 min after induction with mitomycin C, separated on agarose and blotted with a SaPIbov5-specific probe. Upper band is ‘bulk’ DNA, and represents replicating SaPIbov5. SaPI monomer represents SaPI DNA packaged in small capsids.

To identify the ϕ12 gene(s) targeted by Ccm, we isolated ϕ12 mutants insensitive to the Ccm-mediated interference. Four of the mutants were sequenced. All had point mutations in gp33, which corresponds to the ϕ12 major capsid protein (CP) [28], although some of the mutants also had mutations in other genes (table 1). To clearly establish whether ϕ12 gp33 was the target gene of the SaPIbov5 Ccm, we generated a lysogenic RN4220 derivative carrying the phage ϕ12^evolved4^ and the SaPIbov5^adjusted^ island. SOS induction of this strain induced SaPIbov5 replication and transfer (electronic supplementary material, table S6), but not the production of SaPI-sized DNA (figure 6). Moreover, ovexpression of SaPIbov5 Ccm protein from the expression constructs described above caused only a slight reduction of ϕ12^evolved4^ titres (figure 5*b,c*). Taken together, these results confirm that the ϕ12 CP (gp33) was the target for SaPIbov5 Ccm.

**Table 1.** ϕ12 mutants insensitive to the Ccm-mediated interference.

phage	ORF33	ORF45
ϕ12^evolved1^	G3576E; T357S	S13R
ϕ12^evolved2^	T323P	I53S
ϕ12^evolved3^	E236 K	E203 K
ϕ12^evolved4^	E236 K	—

Finally, RN4220 derivatives carrying SaPIbov5 mutants in ORFs 8-12 were infected with ϕ12^evolved4^ and both the phage titre and the plaque sizes were analysed. Based on the results above, we expected this phage to be insensitive to SaPIbov5-mediated interference. However, SaPIbov5 severely blocked ϕ12^evolved4^ reproduction, as it did with the original ϕ12 phage (figure 5*a*), suggesting that other SaPIbov5 genes could have a role in this process, similar to the headful SaPIs described previously [26]. Indeed, the titre of ϕ12^evolved4^ was restored to normal by mutants in either ORF10 or ORF11 (*ccm*) (figure 5*a*), suggesting that ORF10 also plays a role in ϕ12 interference.

### (e) SaPIbov5 Ccm and ϕ12 CP are homologues in sequence but not in function

**Figure 7.**
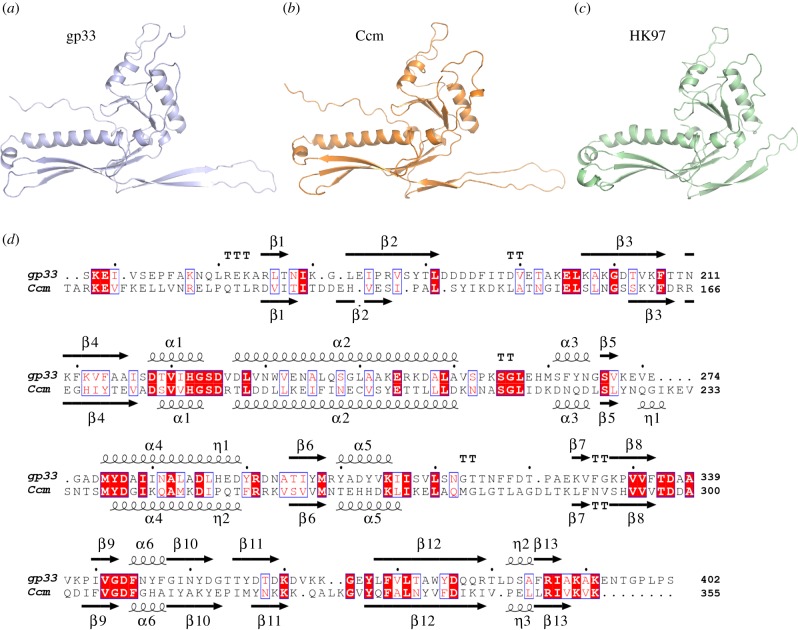
C-terminal portion of gp33 and Ccm proteins are predicted to adopt the characteristic HK97-fold of phage coat proteins. Cartoon representation of the C-terminal portion of (*a*) ϕ12 gp33 (residues 127–402) and (*b*) SaPIbov5 Ccm (residues 83–355), generated by RaptorX [31]. Both proteins show similar folding to the prototypical coat protein from phage HK97 (*c*; PDB 1OHG). (*d*) Structural alignment of ϕ12 gp33 (*a*) and SaPIbov5 Ccm (*b*) models carried out with Mustang [33]. Identical residues are highlighted on a red background and conserved residues are in a blue box with red text. The elements of secondary structure for each model are shown above (gp33) or below (Ccm) the corresponding sequence.

*In silico* analysis of Ccm revealed that this protein has a HK97 major CP-like fold, similar to that of the ϕ12 CP (gp33). In fact, Ccm and the ϕ12 CP seem to be distantly related, based on sequence similarity (figure 7). *In silico* modelling of Ccm and gp33 with RaptorX [31] and Phyre2 [32] servers predicted with high confidence (electronic supplementary material, table S4 and S5) that the C-terminal portions of gp33 (residues 127–402) and Ccm (residues 83–355) both adopt the prototypical coat protein fold from the phage HK97 (figure 7; electronic supplementary material, figure S2) [35,36]. The modelled HK97-fold domains present a high structural similarity both between Ccm and gp33 (RMSD < 1.5 Å for 240 residues) and with HK97 CP (RMSD < 2 Å for 210 residues) despite the low sequence identity (19.2%) (figure 7). By contrast, models with different folds were predicted with low confidence (electronic supplementary material, tables S4 and S5) for the N-terminal portions of Ccm and gp33 proteins (residues 1–82 and 1–126, respectively). However, in all predictions these regions present high α-helical content (electronic supplementary material, figure S3), consistent with the so-called Δ-domain of HK97-like phages, which works as an internal scaffolding protein that assists in CP assembly and is subsequently removed by a phage-encoded protease [36,37].

This putative structural homology raised the interesting possibility that Ccm would be able to form SaPIbov5 capsids in the absence of the ϕ12 CP, suggesting an alternative mechanism to prevent phage reproduction and favouring SaPIbov5 transfer. To address this possibility, we used a previously generated deletion mutant in the gene encoding the CP of ϕSLT (gp42) [28] which is nearly identical to ϕ12 CP (gp33). Next, we introduced the SaPIbov5^adjusted^ island into this strain and measured the phage and transducing titres after SOS induction of the mutant phage. As shown in table 2, ϕSLT CP was essential both for phage and SaPI transfer, showing that Ccm is unable to take the place of the ϕSLT CP.

**Table 2.** Effect of phage mutations on phage and SaPI titres. (The means of results from three independent experiments are shown. Variation was within ±5% in all cases.)

donor strain			
phage	SaPI	phage titre^a^	SaPI titre^b^
ϕ SLT*pvl*::*tet*M	—	5.0 × 10^6^	
ϕ SLT*pvl*::*tet*M ΔORF42	—	<10	—
ϕ SLT*pvl*::*tet*M	SaPIbov5^adjusted^	1.74 × 10^6^	1.72 × 10^6^
ϕ SLT*pvl*::*tet*M ΔORF42	SaPIbov5^adjusted^	<10	<10

^a^PFU ml^−1^ induced culture, using RN4220 as recipient strain.

^b^Number of transductants ml^−1^ induced culture, using RN4220 as recipient strain.

### (f) Ccm blocks *cos* but not *pac* phages

Although conceptually they perform similar functions, *S. aureus cos* and *pac* phages use different proteins for capsid formation and DNA packaging. Thus, we wanted to test whether the reproduction cycle of the *pac* phages was also blocked by the Ccm protein. This was not the case, and expression of the Ccm from plasmid pJP1730 did not block either ϕ11 or 80α reproduction (electronic supplementary material, table S7).

### (g) *Cos* SaPIs reserve space for virulence-gene carriage

SaPIbov2, one of the prototypical *pac* SaPIs [3], is approximately 27 kb in size and cannot redirect the production of small-sized capsids because it does not encode *cpm*B. Consequently, SaPIbov2 is exclusively packaged in large capsids [38]. To know if a similar scenario exists in the *cos* SaPIs, we searched in GenBank for *cos* SaPIs with an increased size and lacking the *ccm* gene. All *cos* SaPIs that were identified encoded Ccm, but one, SaPIS0385, had a reduced size (10.3 kb) compared with the others (electronic supplementary material, figure S4). This island encoded all the genes required for the SaPI cycle, but lacked the classical SaPI-encoded virulence genes. To determine whether a *cos* SaPI with reduced size had a functional ERP cycle, we generated a SaPIbov5 derivative in which the von Willebrand binding protein (*vwb*) and the staphylococcal complement inhibitor (*scn*) genes were deleted (SaPIbov5^small^) (figure 1). The resulting size was 10.9 kb, similar to SaPIS0385 (electronic supplementary material, figure S4). The ϕ12 mediated transfer of the SaPIbov5^small^ element was only slightly reduced (less than twofold) compared with that observed with the wt SaPIbov5. Surprisingly, although the small island expresses the Ccm protein and interferes with ϕ12 reproduction (electronic supplementary material, table S4), it does not produce the characteristic SaPI band (figure 6). Apparently, SaPIbov5^small^ concatemers are packaged more efficiently into the large capsids. This result suggests that during evolution the *cos* SaPIs have reserved approximately 2 kb of DNA space for the carriage of virulence genes.

## 4. Discussion

In this study, we have described packaging of a family of *cos* SaPIs by *cos* helper phages ϕ12 and ϕSLT, and show that these SaPIs interfere with phage production by forming small capsids that are unable to package complete helper phage genomes. This size redirection process is reminiscent of that found in the previously described *pac* SaPIs, where size redirection depends on the two proteins CpmA and CpmB [14], and CpmB acts as an alternative internal scaffolding protein for the small SaPI capsids [20,39]. Here, we have found that size redirection by SaPIbov5 is dependent on the *ccm* gene, which encodes a HK97-like CP homologue.

How Ccm drives the production of small capsids remains unresolved. The HK97-like CP fold predicted for Ccm raises the interesting possibility that this protein could participate in the capsid assembly or even be part of the capsid shell. Even though our experiments have shown that Ccm is unable to form SaPI-sized capsids by itself, the Ccm fold, highly similar to gp33, might enable both proteins to be assembled together. It has been suggested that the length of the N-terminal Δ-domain correlates with capsid size [40,41]. Our models indicate that the Δ-domain of Ccm is 44 residues shorter than that in gp33. Therefore, the inclusion of Ccm during formation of procapsids could conceivably drive the formation of smaller capsids. This proposed mechanism of action also explains why Ccm does not block ϕ11 and 80α *pac* phages, whose capsid proteins lack a Δ-domain and require a separately expressed scaffolding protein for capsid assembly [21]. The SaPIs mobilized by these phages use an alternative scaffolding protein, CpmB, to induce small capsid formation [21,42]. Thus, both Ccm and CpmB proteins drive small capsid formation by mimicking the scaffolding function in the assembly process, representing another example of the SaPIs' capacity for adaptation to their helper phages.

The role of SaPIbov5 ORF10 in this process is unclear. Although deletion of ORF10 had no effect on the SaPIbov5-induced suppression of wild-type ϕ12, it restored reproduction of ϕ12^evolved4^ (figure 5). However, overexpression of ORF10 alone had no effect on either wild-type or evolved ϕ12. Perhaps, ORF10 and Ccm somehow work together to effect the SaPIbov5-mediated interference, a line of reasoning that we will explore in future research.

The production of small capsids is not just a key feature of SaPI biology, but a widespread mechanism of phage interference. The *Enterocococus faecalis* EfCIV583 element also remodels capsid formation, promoting the formation of small capsids [43]. A similar strategy is used by the *Escherichia coli* P4 plasmid, which remodels helper phage P2 capsid formation by the expression of the P4-encoded external scaffolding protein Sid [44]. The proteins involved in these mechanisms share no homology, suggesting that this is a convergent evolutionary strategy that provides a significant advantage in nature.

All the *cos* SaPIs that we have identified encode proteins basically identical to the SaPIbov5 ORFs 8–12 (electronic supplementary material, figure S4), suggesting that all these proteins are involved in the same biological process, being required together to develop their function in the SaPIbov5 cycle. Many of the *pac* SaPIs also encode a variant of the *ppi* gene that is found in *cos* SaPIs, where they act to suppress helper phage DNA packaging. However, the SaPIbov5 *ppi* gene does seem to be involved in *cos* phage ϕ12 interference, which is not surprising, as the terminase enzymes of the *pac* and *cos* site phages are completely different. The function of *ppi* in the SaPIbov5 ERP cycle thus remains unsolved.

SaPIs are widespread elements in nature. Most *S. aureus* strains carry more than one of these elements. SaPIs carry important virulence factors that are unique to these elements and affect the fitness of their bacterial hosts [1,45]. Interestingly, there appears to be little difference in the virulence genes that are carried by the *pac* and *cos* SaPIs. Thus, the genes encoding the TSST-1, Sel or Sec toxins, the staphylococcal complement inhibitor Scin or the von Willebrand factor-binding protein are found in both types of SaPIs. Because of the limited number of chromosomal (*att*C) sites where the SaPIs can integrate and the high number of circulating SaPIs, there is strong competition for the SaPIs to persist. These elements have evolved to carry all the genes required for their own replication and helper phage exploitation, while ‘reserving’ a space for the carriage of virulence genes, which at the end will be essential to compete with other SaPIs. However, the number of genes that can be carried in an SaPI is limited, as an increase beyond the size that can be carried within a small capsid would be absolutely detrimental. Thus, the SaPI-encoded virulence genes should be key for the adaption of *S. aureus* to specific niches or hosts. In support of this, two SaPI-coded genes, *bap* and *vwb*, carried in ruminant *S. aureus* strains, play an important role in the pathogenesis of *S. aureus* in these animal hosts [3,27]. Thus, the identification and blockage of the activity of the SaPI-coded virulence genes can provide novel strategies to combat *S. aureus* infections in a more efficient way. The SaPIs have evolved to exploit and interfere with phage reproduction in a multitude of ways. Other PICIs are likely to use similar strategies. We anticipate that there are many additional mechanisms of interference in SaPIs and other PICIs that remain to be uncovered, and that will be of considerable importance to the evolution of virulence in *S. aureus*.

## Supplementary Material

Supplementary material
