# Supplementary material for: Convergent evolution of pathogenicity islands in helper cos phage interference
Source: Philos Trans R Soc Lond B Biol Sci. 2016 Nov 5;371(1707):20150505. doi: 10.1098/rstb.2015.0505 (PMC5052747; doi:10.1098/rstb.2015.0505)
Supplement: Supplementary material [file rstb20150505supp1.pdf]

Philosophical Transactions B.

Doi: 10.1098/rstb.2015.0505

Supplemental Information

Convergent evolution of pathogenicity islands in helper *cos* phage interference

Nuria Carpena, Keith A. Manning, Terje Dokland, Alberto Marina, José R Penadés.

Table S1. Bacterial strains used in this study.

Strains	Description	Reference
RN4220	Restriction-defective derivative of RN450	[1]
RN451	RN450 lysogenic for ϕ11	[2]
RN10359	RN450 lysogenic for ϕ80α	[3]
JP10435	RN4220 lysogenic for ϕ12	[2]
JP12520	RN4220 lysogenic for ϕ12 evolved 1	This work
JP12521	RN4220 lysogenic for ϕ12 evolved 2	This work
JP12522	RN4220 lysogenic for ϕ12 evolved 3	This work
JP12523	RN4220 lysogenic for ϕ12 evolved 4	This work
JP5011	RN4220 lysogenic for ϕSLT *pvl*::*tet*M	[4]
JP9108	JP5011 ϕSLT *pvl*::*tet*M ΔORF42	[5]
LUG1170	SH1000 ϕSLT	[6]
JP11194	LUG1170 SaPIbov5 *vwb*::*tet*M original	[5]
JP11010	JP10435 SaPIbov5 *vwb*::*tet*M original	[5]
JP12942	JP12523 SaPIbov5 *vwb*::*tet*M original	This work
JP12684	JP10435 SaPIbov5 *vwb*::*tet*M evolved	This work
JP11425	LUG1170 SaPIbov5 *vwb*::*tet*M evolved	This work
JP12685	LUG1170 SaPIbov5::*erm*C adjusted	This work
JP11634	RN4220 SaPIbov5::*erm*C adjusted	This work
JP13161	RN4220 SaPIbov5::*erm*C adjusted ORF8 mutant	This work
JP12648	RN4220 SaPIbov5::*erm*C adjusted ORF9 mutant	This work
JP13048	RN4220 SaPIbov5::*erm*C adjusted ORF10 mutant	This work
JP13087	RN4220 SaPIbov5::*erm*C adjusted ORF11 mutant	This work
JP12618	RN4220 SaPIbov5::*erm*C adjusted ORF12 mutant	This work
JP13192	RN4220 SaPIbov5::*erm*C adjusted ORF8 to12 mutant	This work
JP12419	JP10435 SaPIbov5::*erm*C adjusted	This work
JP13171	JP10435 SaPIbov5::*erm*C adjusted ORF8 mutant	This work
JP12688	JP10435 SaPIbov5::*erm*C adjusted ORF9 mutant	This work
JP13162	JP10435 SaPIbov5::*erm*C adjusted ORF10 mutant	This work
JP13110	JP10435 SaPIbov5::*erm*C adjusted ORF11 mutant	This work
JP12636	JP10435 SaPIbov5::*erm*C adjusted ORF12 mutant	This work
JP13298	JP10435 SaPIbov5::*erm*C adjusted ORF8 to 12 mutant	This work
JP13443	JP13298 pJP1769	This work
JP11905	JP5011 SaPIbov5::*erm*C adjusted	This work
JP11906	JP5011 ϕSLT *pvl*::*tet*M ΔORF42 SaPIbov5::*erm*C adjusted	This work

Strains	Description	Reference
JP12736	JP10435 SaPIbov5::*erm*C small	This work
JP12867	JP12523 SaPIbov5::*erm*C small	This work
JP12686	LUG1170 SaPIbov5::*erm*C small	This work
JP12380	RN4220 pCN51	This work
JP12945	RN4220 pJP1749	This work
JP12800	RN4220 pJP1748	This work
JP12968	RN4220 pJP1747	This work
JP11423	RN4220 pJP1730	This work
JP12647	RN4220 pJP1745	This work
JP11367	JP5011 pCN51	[5]
JP11559	JP5011 pJP1730	This work
JP12293	RN10359 pCN51	This work
JP11556	RN10359 pJP1730	This work
JP12343	RN451 pCN51	This work
JP11557	RN451 pJP1730	This work
JP12294	JP10435 pCN51	This work
JP13006	JP10435 pJP1749	This work
JP13007	JP10435 pJP1748	This work
JP13008	JP10435 pJP1747	This work
JP11558	JP10435 pJP1730	This work
JP13009	JP10435 pJP1745	This work
JP13012	JP12523 pCN51	This work
JP13013	JP12523 pJP1749	This work
JP13014	JP12523 pJP1748	This work
JP13015	JP12523 pJP1747	This work
JP13016	JP12523 pJP1730	This work
JP13017	JP12523 pJP1745	This work

Table S2. Plasmids used in this study.

Plasmid	Description	Reference
pBT$_2$-βgal	Vector for efficient allelic replacement	Lab plasmid
pJP1712	pBT$_2$-βgal derivative, insertion of the *erm*C cassette in SaPIbov5^{adjusted}	This work
pJP1761	pBT$_2$-βgal derivative, insertion of the *erm*C cassette in SaPIbov5^{small}	This work
pJP1757	pBT$_2$-βgal derivative, ochre mutation in SaPIbov5 ORF8	This work
pJP1758	pBT$_2$-βgal derivative, ochre mutation in SaPIbov5 ORF9	This work
pJP1759	pBT$_2$-βgal derivative, ochre mutation in SaPIbov5 ORF10	This work
pJP1717	pBT$_2$-βgal derivative, ochre mutation in SaPIbov5 ORF11	This work
pJP1760	pBT$_2$-βgal derivative, ochre mutation in SaPIbov5 ORF12	This work
pCN51	Derivative of pCN51. Expression vector	[7]
pJP1749	pCN51-*ppi* SaPIbov5	This work
pJP1748	pCN51-*orf9* SaPIbov5	This work
pJP1747	pCN51-*orf10*SaPIbov5	This work
pJP1730	pCN51-*ccm* SaPIbov5	This work
pJP1745	pCN51-*orf12* SaPIbov5	This work
pCN51	Derivative of pCN51 with chloramphenicol cassette. Expression vector	This work
pJP1769	pCN51-*cat194-ccm* SaPIbov5	This work

Table S3. Primers used in this study.

Plasmid	Oligonucleotides	Sequence (5'-3')
pJP1712	SaPIbov5-29mE	CCGGAATTCGCATAATGACTACCGCATTAG
	SaPIbov5-30cB	CGCGGATCCATTTTTCATTACCATTAAATAAATTTATATACCTG
	SaPIbov5-31mPst1	AACTGCAGAAATTAATAAGGTGTATAACGGTAAATAG
	SaPIbov5-32sEcoR5	AAGATATCGTAATCATAACATAAAAGCACCCC
pJP1761	SaPIbov5-57mE	CCGGAATTCTATGTAAAGGTGTGGTTATTTGG
	SaPIbov55-58cB	CGCGGATCCCTATGAAAGTTCTTTTTTAACTTGT
	SaPIbov5-59mPst1	AACTGCAGGTTATGATATGTTATACATGCATCA
	SaPIbov5-60cEcoR5	AAGATATCAATGAAATAAGTATTTTTCGTTAGTTTG
pJP1757	SaPIbov5-90mB	CGCGGATCCTTCAGTCACACCTATAAACACTG
	SaPIbov5-91c	TTTGAATTCTTGTTCTTAAAATACATT
	SaPIbov5-92m	AATGTATTTTAAGAACAAGAATTCAAA
	SaPIbov5-93cS	ACGCGTCGACTCATTTGTATGATCTAGCACGTC
pJP1758	SaPIbov5-78mB	CGCGGATCCGAAGAACTTGCAGTTACTCAAAA
	SaPIbov5-75c	GATTATTTGGAATTCGATTCTTTAATGATT
	SaPIbov5-76m	AATCATTAAAGAATCGAATTCCAAATAATC
	SaPIbov5-77cS	ACGCGTCGACAAAGTTAGATAGTTGGTACCCAA
pJP1759	SaPIbov5-81mB	CGCGGATCCAAGGTCTAAGGCTACCAATTGT
	SaPIbov5-94c	TACGTTCGTTGTTTTGAATTCTTCTTAGAACGAAATGTTA
	SaPIbov5-95m	TAACATTTCGTTCTAAGAAGAATTCAAAACAACGAACGTA
	SaPIbov5-96cS	ACGCGTCGACGATAGCTCAATACCATTAGTAGCTA
pJP1717	SaPIbov5-97mB	CGCGGATCCGTAAGGCGCTATACAAAAAAGC
	SaPIbov5-98c	TCTCACTACTCACTAAGAATTCCTGTTTATGTACCTACCCATG
	SaPIbov5-99m	CATGGGTAGGTACATAAACAGGAATTCTTAGTGAGTAGTGAGA
	SaPiBov5-100cS	ACGCGTCGACCAACAGATACTTTTCGTCTAAACG
pJP1760	SaPIbov5-65mB	CGCGGATCCCGTTTAGACGAAAAGTATCTGT
	SaPIbov5-66c	ATTGTAGAATTCTAAAAGTTCTACTTAAGTGATTTTATC
	SaPIbov5-67m	GATAAAATCACTTAAGTAGAACTTTTAGAATTCTACAAT
	SaPIbov5-68cS	ACGCGTCGACTAACTCCTCATCATCTCCGTCC
pJP1749	SaPIbov5-78mB	CGCGGATCCGAAGAACTTGCAGTTACTCAAAA
	SaPIbov5-87cXma1	CCCCCCGGGTTAATTTACTAAACTAAATGTTACTG
pJP1748	SaPIbov5-81mB	CGCGGATCCAAGGTCTAAGGCTACCAATTGT
	SaPIbov5-82cE	CCGGAATTCTCATTTGTATGATCTAGCACGTC
pJP1747	SaPIvov5-80mB	CGCGGATCCGTATAGATATGGGAGTGTGTATATT
	SaPiBov5-88cXma1	CCCCCCGGGTTAAACTCTTTCATAATCTTCATCTC
pJP1730	SaPIbov5-54mB	CGCGGATCCTTTCTTCATAACATTTCGTTCTATGA
	SaPIbov5-55cE	CCGGAATTCTTATTTAACCTTAACGATTCTCAGTA
pJP1745	SaPIbov5-64mB	CGCGGATCCGCAACGCGGATAAACTTTTATG
	SaPIbov5-56cE	CCGGAATTCCTATGAAAGTTCTTTTTTAACTTGT
pJP1769	SaPIbov5-54mB	CGCGGATCCTTTCTTCATAACATTTCGTTCTATGA
	SaPIbov5-55cE	CCGGAATTCTTATTTAACCTTAACGATTCTCAGTA
SaPIbov5 Southern blot probe	SaPIbov1-112mE	CGCGGATCCTAATTCTCCACGTCTAAAGC
	SaPIbov1-113cB	CCGGAATTCAATTGCTGAGGCAAAACTTC

Table S4. Templates and confidence values in gp33 and Ccm models generated by Phyre2 server[a].

	Protein	Template information	PDB code	Aligned Residues[a]	Confidence[b] (%)	Identity (%)
C-terminal portion	gp33	Capsid protein form bacteriophage HK97	2FT1	132-388	100	18
		Capsid protein form bacteriophage CW02	3J1A	211-388	99.6	25
		Capsid protein form bacteriophage BPP-1	3J4U	148-338	98.2	17
		Thermotoga maritima encapsulin protein (TM-0786)	3DKT	147-392	97.9	15
		Capsid protein form bacteriophage T7	3J7W	135-387	97.8	15
		Myxococcus xanthus encpasulin protein (EncA)	4PT2	147-388	97.6	11
		Capsid protein form marine bacteriophage Syn5	4BML	144-387	97.6	15
		Virus-like particule from *Pyrococcus furiosus*	2E0Z	147-392	97.4	9
	Ccm	Capsid protein form bacteriophage HK9	2FT1	153-349	99	15
		Capsid protein form bacteriophage HK97	1IF0	159-349	98.9	14
		Capsid protein form bacteriophage HK97	3P8Q	153-341	97.9	15
		Capsid protein form bacteriophage CW02	3J1A	186-309	76.8	26
		Capsid protein form bacteriophage BPP-1	3J4U	234-299	74.7	20
		Capsid protein form bacteriophage HK97	2FT1	153-349	99	15
N-terminal portion	gp33	Coiled-Coil Protein of Unknown Function from *Eubacterium eligens*	3HNW	19-84	93.3	12
		Human Fibrinogen beta chain	3GHG	17-87	84.4	19
		Anopheles Plasmodium-responsive Leucine-rich repeat protein 1 (LRR1)	3OJA	17-90	84.3	12
		Bovine Fibrinogen beta chain	1DEQ	20-91	84.1	13
		Human cytoplasmic dynein 2 heavy chain	4RH7	17-119	83.6	10
		PcsB protein from *Streptococcus pneumoniae*	4CGK	17-82	79.4	13
		Seryl-tRNA synthetase N-terminal domain from *Aquifex aeolicus*	2DQ3	14-91	78.8	13
		dynein motor heavy chain	3VKG	1-84	67.5	5
	Ccm	Mit domain-containing protein 1	2YMB	109-157	14.9	20
		Human Striatin-3 coiled coil domain	4N6J	31-47	14.8	35

[a]See reference [8] for details.
[b]Aligned residues. Part of protein sequence that is aligned with the template sequence.
[c]Confidence represents the probability (from 0 to 100) that the match between the query sequence and the corresponding template is a true homology. A match with confidence >90%, generally should indicate that the query sequence adopts the overall fold shown by the template and that the core of the protein is modelled at high accuracy.

Table S5. Templates and confidence values in gp33 and Ccm models generated by RaptorX server[a].

	Protein	Template information	PDB code	P-value[b]	Score[c]	uGDT/ GDT[d]	uSeqID/ SeqID[e]
C-terminal portion	gp33	Capsid protein form bacteriophage HK97	1OHG	3.1 e-08	149	147/57	42/16
		Capsid protein form bacteriophage HK97	3QPR	2.1 e-05	99	121/51	31/13
		Thermotoga maritima encapsulin protein (TM-0786)	3DKT	1.5 e-04	83	72/31	27/12
		Myxococcus xanthus encpasulin protein (EncA)	4PT2	3.0 e-04	78	51/29	10/6
		Virus-like particule from *Pyrococcus furiosus*	2E0Z	1.0 e-04	86	60/27	18/8
	Ccm	Capsid protein form bacteriophage HK97	1OHG	9.1 e-08	133	144/55	29/11
		Capsid protein form bacteriophage HK97	3QPR	2.2 e-05	93	94/47	18/9
		Thermotoga maritima encapsulin protein (TM-0786)	3DKT	5.9 e-05	85	72/30	26/11
		Myxococcus xanthus encpasulin protein (EncA)	4PT2	2.3 e-04	75	50/28	12/7
		Virus-like particule from Pyrococcus furiosus	2E0Z	6.7 e-05	84	56/26	22/10
N-terminal portion	gp33	Structural maintenance of chromosomes protein 4 (Smc4) from Yeast	4RSI	1.1 e-02	61	52/47	13/12
		CT398 protein from *Chlamydia trachomatis*	4ILO	1.0 e-02	62	52/47	12/11
		HP0958 Protein from *Helicobacter pylori*	3NA7	1.1 e-02	60	52/47	18/16
		Colicin IA from *Escharichia coli*	1CII	8.0 e-03	64	41/43	8/9
		Cytoplasmic dynein 1 heavy chain 1 from *Mus musculus*	3WUQ	9.9 e-03	62	49/44	15/14
	Ccm	iSH2 domain of human phosphatidylinositol 3-kinase p85beta	3MTT	2.7 e-02	28	42/53	10/12
		Vacuolar protein sorting-associated protein 30	5DFZ	1.8 e-02	30	42/52	8/10
		PcsB protein from *Streptococcus pneumoniae*	4CGK	2.9 e-02	27	42/52	10/12
		HP0958 Protein from *Helicobacter pylori*	3NA7	2.9 e-02	28	42/52	8/10
		Autophagy protein Apg17	4HPQ	3.1 e-02	27	41/53	5/6

[a]See reference [9] for details.
[b]P-value. Likelihood of a predicted model being worse than the best of a set of randomly-generated models. For mainly alpha proteins, P-value < e-3 and e-4 are good indicator for mainly alpha and beta proteins, respectevely.
[c]Score. The alignment score falling between 0 and the (domain) sequence length, with 0 indicating the worst.
[d]uSeqID and SeqID. uSeqID is the number of identical residues in the alignment. SeqID is uSeqID normalized by the protein (or domain) sequence length and multiplied by 100.
[e]uGDT and GDT. uGDT is the unnormalized GDT (Global Distance Test) score defined as 1*N(1) + 0.75 * N(2) + 0.5 * N(4) + 0.25 * N(8), where N(x) is the number of residues with estimated modeling error (in Å) smaller than x. GDT is calculated as uGDT divided by the protein (or domain) length and multiplied by a 100.

Table S6. Effect of SaPIbov5 size on phage and SaPI titres[a].

Donor strain			
Phage	SaPI	Phage titre[b]	SaPI titre[c]
ϕ12	-	1.9×10^{10}	-
ϕ12	SaPIbov5^{original}	2.0×10^{5}	3.09×10^{5}
ϕ12	SaPIbov5^{evolved}	1.9×10^{6}	6.8×10^{6}
ϕ12	SaPIbov5^{adjusted}	3.3×10^{6}	1.33×10^{7}*
ϕ12	SaPIbov5^{small}	1.5×10^{6}	1.8×10^{6}
ϕ12 evolved 4	-	9.2×10^{9}	-
ϕ12 evolved 4	SaPIbov5^{original}	1.2×10^{6}	2.4×10^{4}
ϕ12 evolved 4	SaPIbov5^{adjusted}	4.7×10^{6}	1.1×10^{7}*
ϕ12 evolved 4	SaPIbov5^{small}	5.4×10^{6}	1.3×10^{6}
ϕ SLT	-	6.6×10^{7}	-
ϕ SLT	SaPIbov5^{original}	8.2×10^{5}	7.5×10^{3}
ϕ SLT	SaPIbov5^{evolved}	1.0×10^{6}	1.16×10^{6}*
ϕ SLT	SaPIbov5^{adjusted}	1.1×10^{6}	2.7×10^{6}*
ϕ SLT	SaPIbov5^{small}	8.2×10^{5}	1.7×10^{5}

[a]The means of results from three independent experiments are shown. Variation was within ±5% in all cases.
[b]PFU/ml induced culture, using RN4220 as recipient strain
[c]No. of transductants/ml induced culture, using RN4220 as recipient strain.
*Significant at $p<0.05$, using Kruskal-Wallis with Dunn's Multiple Comparison Test probabilities.

Table S7. Role of Ccm in phage interference.

Phage infection

Phage	No. phage plaques	
	RN4220[b]	RN4220 + pCN51-*ccm*[b]
80α	2.0×10^{12}	8.0×10^{12}
ϕ11	5.2×10^{11}	1.6×10^{12}
ϕ12	2.8×10^{10}	1.4×10^{7}
ϕSLT*pvl*::*tet*M	8.8×10^{6}	1.0×10^{2}

Phage induction

Donor Strain		Phage titre
Lysogen	Plasmid	RN4220[b]
80α	pCN51	7.0×10^{10}
80α	pCN51-*ccm*	1.0×10^{10}
ϕ11	pCN51	5.0×10^{9}
ϕ11	pCN51-*ccm*	1.4×10^{9}
ϕ12	pCN51	1.8×10^{10}
ϕ12	pCN51-*ccm*	1.1×10^{6}
ϕSLT*pvl*::*tet*M	pCN51	1.7×10^{5}
ϕSLT*pvl*::*tet*M	pCN51-*ccm*	6.0×10^{2}

[a]The means of results from three independent experiments are shown. Variation was within ±5% in all cases.
[b]PFU/ml induced culture, using RN4220 as recipient strain.

References

1. Kreiswirth, B. N., Löfdahl, S., Betley, M. J., O'Reilly, M., Schlievert, P. M., Bergdoll, M. S. & Novick, R. P. 1983 The toxic shock syndrome exotoxin structural gene is not detectably transmitted by a prophage. *Nature* **305**, 709–712.

2. Novick, R. 1967 Properties of a cryptic high-frequency transducing phage in *Staphylococcus aureus*. *Virology* **33**, 155–166.

3. Ubeda, C., Barry, P., Penadés, J. R. & Novick, R. P. 2007 A pathogenicity island replicon in *Staphylococcus aureus* replicates as an unstable plasmid. *Proc. Natl. Acad. Sci. U.S.A.* **104**, 14182–14188. (doi:10.1073/pnas.0705994104)

4. Ferrer, M. D. et al. 2011 RinA controls phage-mediated packaging and transfer of virulence genes in Gram-positive bacteria. *Nucleic Acids Res.* **39**, 5866–5878. (doi:10.1093/nar/gkr158)

5. Quiles-Puchalt, N., Carpena, N., Alonso, J. C., Novick, R. P., Marina, A. & Penadés, J. R. 2014 Staphylococcal pathogenicity island DNA packaging system involving *cos*-site packaging and phage-encoded HNH endonucleases. *Proc. Natl. Acad. Sci. U.S.A.* **111**, 6016–6021. (doi:10.1073/pnas.1320538111)

6. Labandeira-Rey, M. et al. 2007 *Staphylococcus aureus* Panton-Valentine leukocidin causes necrotizing pneumonia. *Science* **315**, 1130–1133. (doi:10.1126/science.1137165)

7. Charpentier, E., Anton, A. I., Barry, P., Alfonso, B., Fang, Y. & Novick, R. P. 2004 Novel cassette-based shuttle vector system for gram-positive bacteria. *Appl. Environ. Microbiol.* **70**, 6076–6085. (doi:10.1128/AEM.70.10.6076-6085.2004)

8. Kelley, L. A., Mezulis, S., Yates, C. M., Wass, M. N. & Sternberg, M. J. E. 2015 The Phyre2 web portal for protein modeling, prediction and analysis. *Nat Protoc* **10**, 845–858. (doi:10.1038/nprot.2015.053)

9. Källberg, M., Wang, H., Wang, S., Peng, J., Wang, Z., Lu, H. & Xu, J. 2012 Template-based protein structure modeling using the RaptorX web server. *Nat Protoc* **7**, 1511–1522. (doi:10.1038/nprot.2012.085)

```
phiSLT          tatgcccccctgcccatcggcttaaaatgttttttcgccgggtaccggagaggcccaaac
phi12           tatagccccctacccatcggcttaaaatgttttttcgacgggtaccggcgggggcccttc
SaPIbov5        tatagccccctgcccattggcttaaaatgttttttcgccgggtaccggcgggggcccttc
                ***. ******.*****.******************* ********** *.** **   *

phiSLT          gctagcaacgcggataaatttttcatgaaaggggg
phi12           gcttgcaacgcggataaactttt-atgaaaggggg
SaPIbov5        gcttgcaacgcggataaactttt-atgaaaggggg
                *** **************.**** ***********
```

Figure S1. *Cos* alignment. The SaPIbov5 and phage *cos* sites and their flanking sequences are aligned using MUSCLE. The *cos* sites are shaded in yellow.

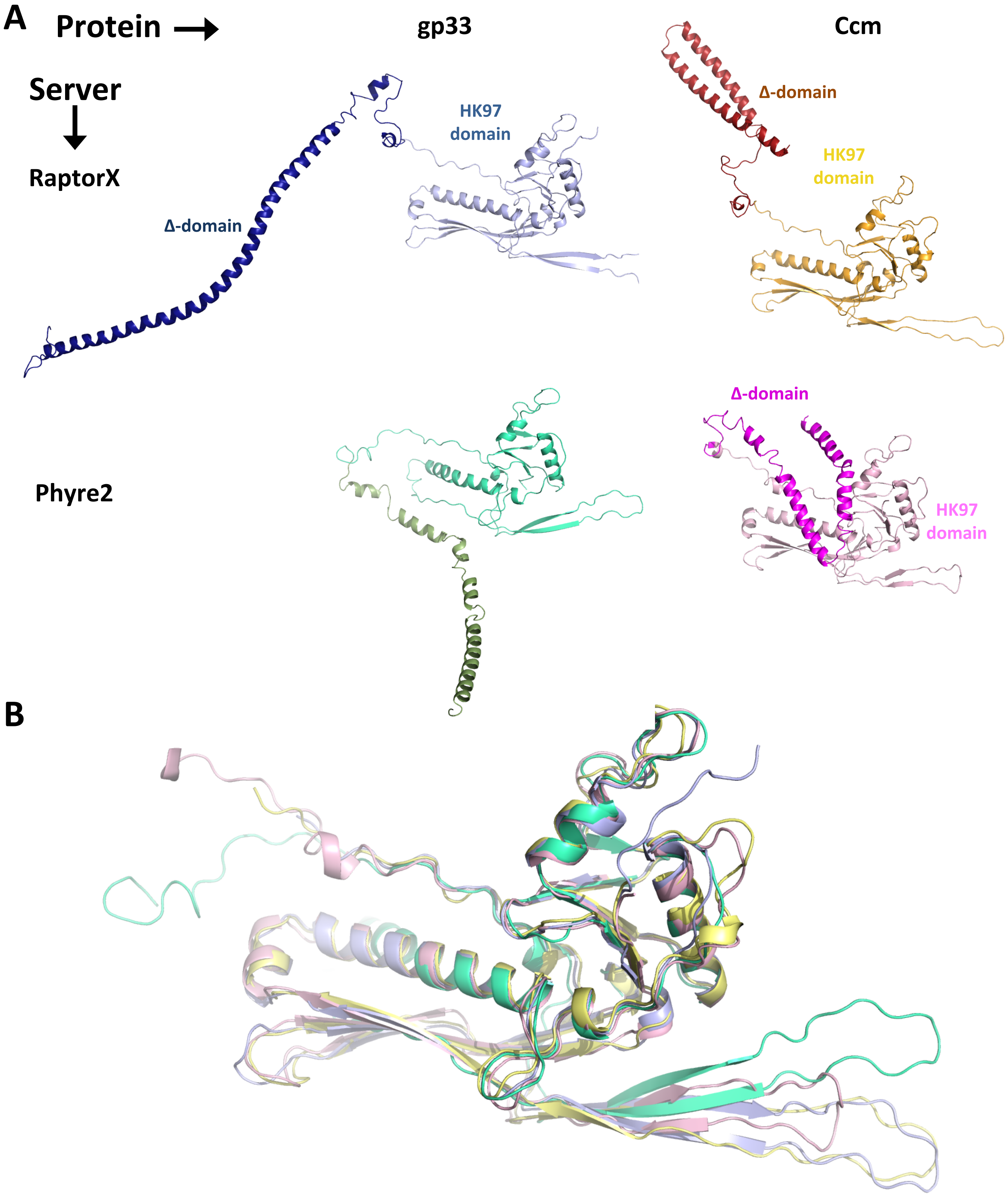

Figure S2. Comparison of gp33 and Ccm structural models generated by RaptorX and Phyre2 Servers. (*a*) Cartoon representation of the structural models of gp33 and Ccm proteins produced by RaptorX and Phyre2 servers (see material and methods for details). Both servers divided the proteins into two portions: a N-terminal portion (highlighted in dark hue) with high α-helical content characteristic of coat protein Δ-domains and a C-terminal portion (light hues) showing the HK97-fold. (*b*) Superimposition of C-terminal portions for the four models (two for each protein). The four models (identical colours than A) agree in that gp33 and Ccm present an structural similar C-terminal portion with HK97-fold.

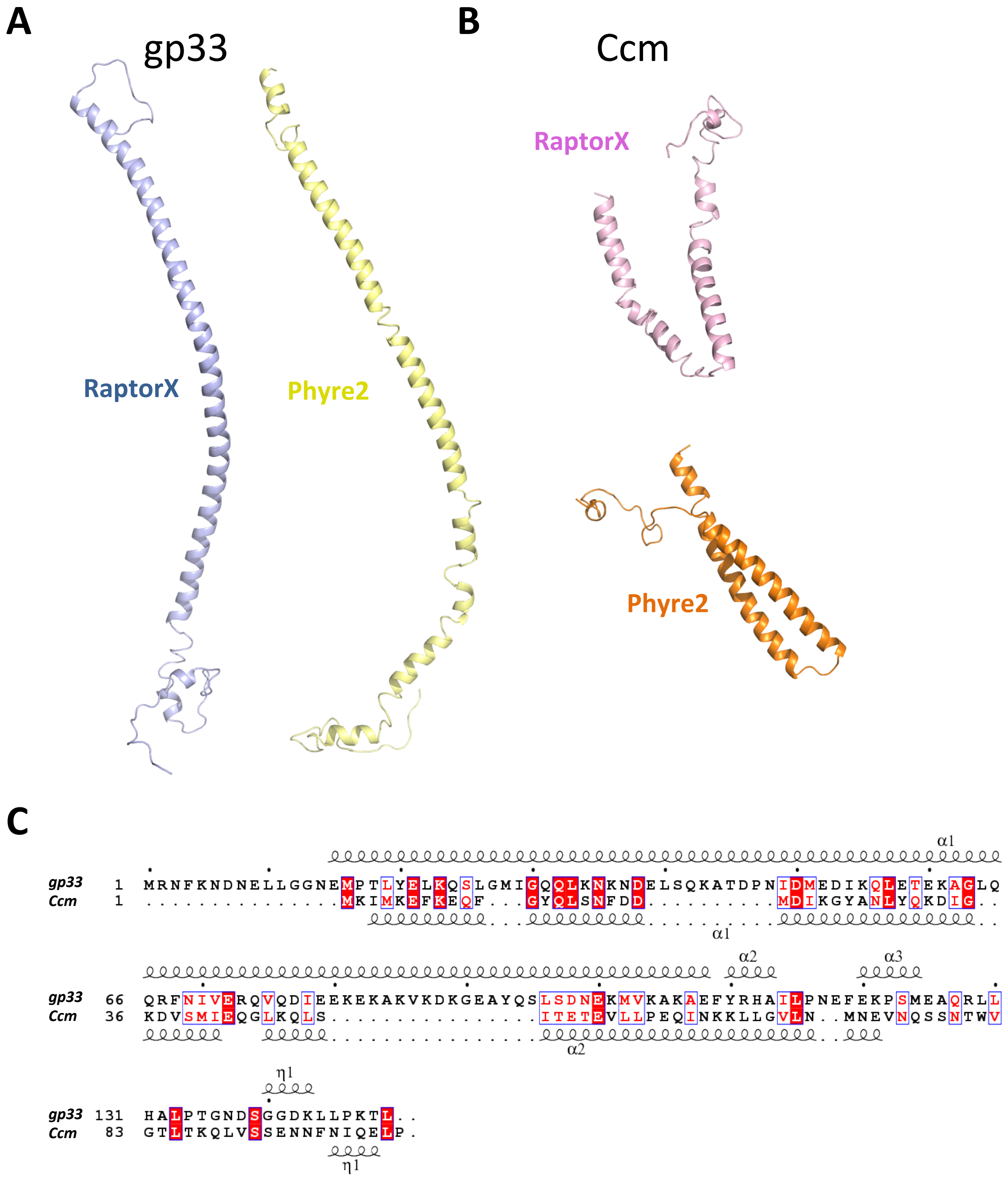

Figure S3. N-terminal portion of gp33 and Ccm are predicted to adopt an α-helical fold. Cartoon representation of the predicted models by RaptorX and Phyre2 serves for the N-terminal portion of (*a*) gp33 (residues 1-126) and (*b*) Ccm (residues 1-82). (*c*) Structural alignment of gp33 and Ccm models generated by RaptorX carried out with Mustang. Identical residues are highlighted on a red background and conserved residues are in a blue box with red text. The elements of secondary structure for each model are shown above (gp33) or below (Ccm) the corresponding sequence.

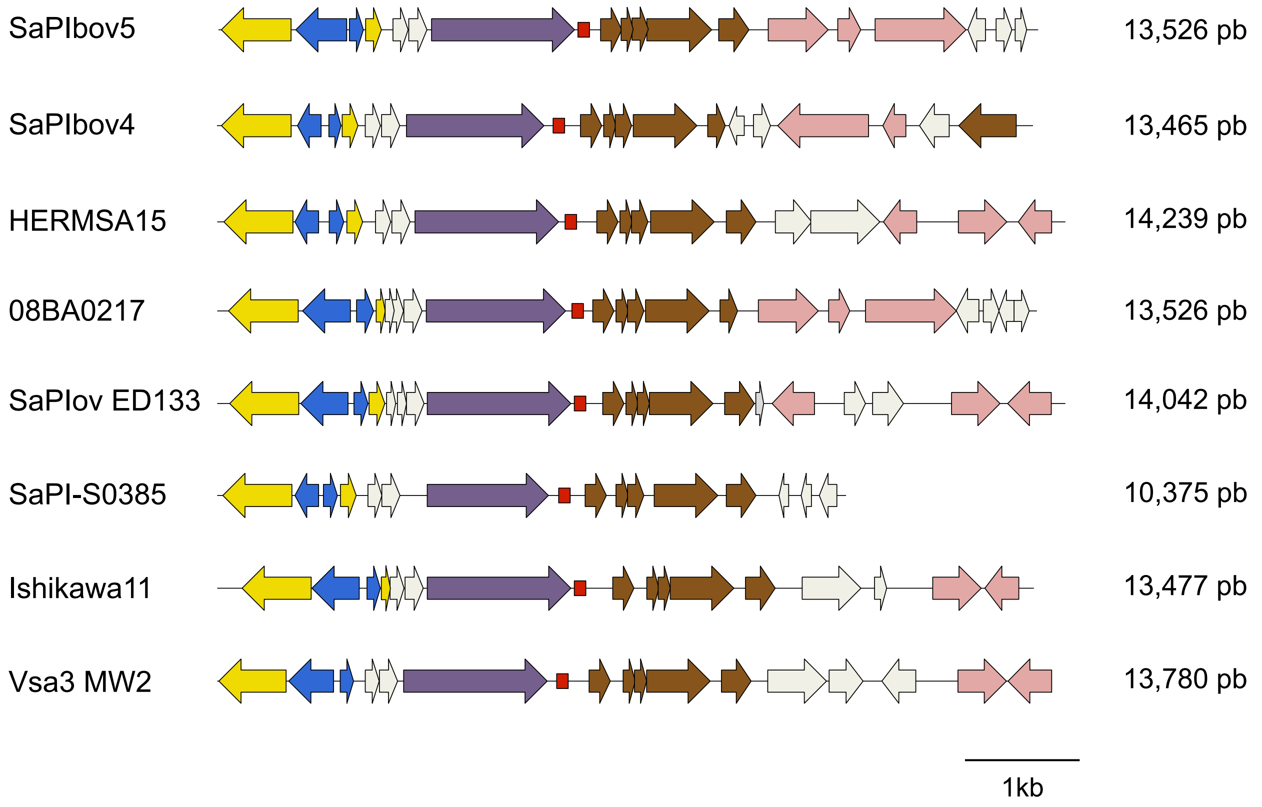

Figure S4. Comparison of a subset of phage-inducible chromosomal island genomes. Genomes are aligned according to the prophage convention, with the integrase gene (*int*) at the left end. Genes are colored according to their sequence and function: *int* and *xis* (excisionase) are yellow; SaPI transcription regulators (*stl, str*) are blue; the replication genes primase (*pri*) and replication initiator (*rep*) are purple; the replication origin (*ori*), red; genes of the new morphogenetic operon are brown; superantigen and other virulence genes are pink. Genes encoding hypothetical proteins are white.
